# The role of immunity in mosquito-induced attenuation of malaria virulence

**DOI:** 10.1186/1475-2875-13-25

**Published:** 2014-01-21

**Authors:** Margaret J Mackinnon

**Affiliations:** 1 KEMRI-Wellcome Trust Research Programme, PO Box 230, Kilifi, Kenya; 2 Centre for Tropical Medicine, Nuffield Department of Medicine, University of Oxford, Oxford OX3 7LJ, UK

**Keywords:** Malaria, Virulence, Mosquito transmission, Immunity

## Abstract

A recent study found that mosquito-transmitted (MT) lines of rodent malaria parasites elicit a more effective immune response than non-transmitted lines maintained by serial blood passage (non-MT), thereby causing lower parasite densities in the blood and less pathology to the host. The authors attribute these changes to higher diversity in expression of antigen-encoding genes in MT cf. non-MT lines. Alternative explanations that are equally parsimonious with these new data, and results from previous studies, suggest that this conclusion may be premature.

## Background

Many studies have shown that when parasite lines of the rodent malaria *Plasmodium chabaudi* or other malaria species are transmitted through mosquitoes, their virulence and asexual replication rate in the blood is reduced [1-6]. This phenomenon is most obvious when lines have been maintained in the laboratory by serial blood passage (SBP) without mosquito transmission: this is because continuous serial passage leads to progressively higher virulence and replication rates [1,6-13]. Spence *et al.*[6] have recently re-visited the phenomenon of mosquito-induced virulence attenuation, in particular addressing whether it alters the parasite’s interaction with the immune system of its host. They found that MT parasites induce a less effective immune response than their non-MT counterparts, and further found differences between MT and non-MT lines in the expression of a set of putative antigen-encoding genes.

### Interactions between evolved virulence, immunity and mosquito transmission

The family of genes that Spence *et al*. [6] found to be differentially expressed between MT and non-MT lines encode a large, highly variable set of antigens thought to be displayed on the surface of the parasite-infected red cell where they interact with the immune system. Non-MT lines expressed a few members of this gene family at high frequency and the remainder at low frequency whereas MT lines expressed all members approximately equally. Spence *et al.*’s [6] observation implies that during mosquito transmission the expression pattern of the antigenic repertoire is re-set, thereby restoring antigenic diversity and reducing the dominance of certain antigenic types that arise during serial blood passage.

Does this resetting render MT parasites more controllable by the immune system, or is it just that MT parasites are less intrinsically virulent? In previous experiments studying the evolution of parasite virulence in the same experimental model used by Spence *et al.* (*Plasmodium chabaudi* in C57/Bl6 laboratory mice) parasite lines were evolved by SBP through either immunized or naïve mice and, at the end, virulence of the evolved lines was measured, both in immunized and naïve mice, and both before and after mosquito transmission [1]. Before mosquito transmission, parasites evolved in immunized mice (‘I-lines’) were found to be more virulent and faster replicating than those evolved in naïve mice (‘N-lines’). This held whether or not virulence was measured in naïve or immunized mice. After mosquito transmission, I-lines were still more virulent and faster replicating than N-lines when measured in naïve mice. These results suggested that immune selection had generated higher levels of intrinsic virulence and replication, and that the changes were genetically ‘hard-wired’.

However, when the I-lines were compared to the N-lines in pre-immunized mice, the differences disappeared. In the light of new data from Spence *et al.*[6], it is hypothesized here that selection in immunized mice favours parasites with rare antigenic types. When expression of the antigenic repertoire is re-set in mosquitoes, as found by Spence *et al.*[6], these rare types would switch off, or down, their expression. This would thus eliminate the I-lines’ replication advantage and higher virulence when measured in pre-immunized mice. If it is assumed that these rarer antigenic types were selectively favoured in immunized mice because they were more invisible to the immune system of a non-immunized mouse, these data fit well with Spence *et al*.’s [6] interpretation that mosquitoes induce changes to the way the parasite is ‘seen’ by the immune system.

However, several alternative explanations that invoke changes to the parasite’s intrinsic virulence, as opposed to the immunity it provokes, are similarly parsimonious with the data of Spence *et al.*[6] and from previous experiments [1,2]. Severe population bottlenecking during mosquito passage will eliminate most new high-virulence mutants expected to accumulate during SBP. As theory predicts [14] and experiments have shown [2,15], bottlenecking causes a general, albeit variable, reduction in virulence. Alternatively, SBP might create sub-populations of ‘short-sighted, dead-end’ [16] virulence mutants which grow fast but cannot transmit. The presence of such mutants would not be detected in a mixed population of predominantly transmissible parasites. However, weeding them out during transmission would cause a reduction in virulence, as observed.

## Conclusions

Although virulence attenuation in laboratory models of malaria [1-6] is highly artificial since all natural malaria infections are transmitted via the mosquito, the Spence *et al.*[6] study takes us one step closer towards a molecular understanding of malaria parasite virulence. Just how their observed molecular changes translate to reduced virulence is still open to debate. Delineating among competing explanations is critical because of mounting evidence in *Plasmodium falciparum*, the most deadly of the human malaria parasites, that certain subsets of the variable surface antigens are highly pathogenic [17-20] thus creating hope for an ‘anti-virulence’ vaccine.

## Competing interests

The author declares that she has no competing interests.
